# Effect of acupressure on constipation in patients undergoing hemodialysis: A randomized double-blind controlled clinical trial 

**Published:** 2019

**Authors:** Parivash Abbasi, Mohammad Mojalli, Mojtaba Kianmehr, Somayeh Zamani

**Affiliations:** 1 *Student Research Committee,* *Gonabad University of Medical Sciences, Gonabad, Iran*; 2 *Social Development and Health Promotion Research Center * *&* * Department of Nursing, Faculty of Nursing and Midwifery, Gonabad University of Medical Sciences, Gonabad, Iran *; 3 *Department of Medical Physics, Faculty of Medicine, Gonabad University* * of Medical Sciences, Gonabad, Iran*; 4 *Department of Nutrition, Faculty of Medicine, Mashhad University of Medical Sciences, Mashhad, Iran*

**Keywords:** Acupressure, Hemodialysis, Constipation

## Abstract

**Objective:**

Constipation is one of the most common digestive problems in patients undergoing hemodialysis. It has a negative effect on quality of life in these patients. As routine treatments are not effective in this regard, complementary therapies may help to overcome this condition. This study aimed to investigate the effect of acupressure on constipation in patients undergoing hemodialysis.

**Materials and Methods:**

This was a randomized double- blind placebo- controlled clinical trial conducted in 2014. A convenience sample of 70 patients undergoing hemodialysis was selected from hemodialysis units of three hospitals affiliated to Mazandaran University of Medical Sciences, Mazandaran, Iran. Patients were randomly assigned to intervention or control group. Intervention group received acupressure in acupressure points three times a week for four weeks during hemodialysis. In control group, acupressure was delivered in false points. We assessed the frequency of defecation in the two groups before and after the study. The study instruments consisted of a demographic questionnaire, and a data sheet for documenting constipation frequency.

**Results:**

The results indicated a significant difference between intervention group (13.73±3.63) and control group (10.06±3.77) in frequency of defecation during the fourth week of intervention (p<0.001). Regarding quality of stool, there was a meaningful difference between the groups in the fourth week in a way that the stool in the intervention group was more natural and in the control group, it was thicker and more adhesive.

**Conclusion:**

Acupressure seems to be an effective complementary treatment for constipation in patients undergoing hemodialysis.

## Introduction

One of the most common chronic diseases is chronic renal failure (Atashpeikar et al., 2012 ▶). The number of patients with end-stage renal disease (ESRD) who need dialysis, is increasing in Iran (Vatarii, 2011 ▶). Patients undergoing hemodialysis, complain from chronic constipation so that the prevalence of this disorder has been reported to be 40-53% among these patients (Hammer et al., 1998 ▶; Murtagh et al., 2007 ▶). Constipation is characterized by sustained and difficult defecation, infrequent bowel movements, or incomplete evacuation (Andrews and Storr, 2011 ▶). Dietary restriction of high potassium fruits and vegetables, decreased fiber content in foods, low fluids intake, inactivity and medications such as aluminum and calcium phosphorous binders, iron supplements, and narcotics can cause constipation in patients undergoing hemodialysis (Lew et al., 2001 ▶).

Chronic constipation can cause serious complications such as diverticulosis in colon and melanosis coli (Stark, 1999 ▶). It affects physical activities and psychological wellbeing and further consequences are reduced quality of life and high economical burden for both families and society (Rogers, 2011 ▶). However, constipation exacerbates hyperkalemia in these patients, because 30-40 meq of potassium is excreted in feces daily (Lehnhardt and Kemper, 2011 ▶). Consumption of laxatives and oral anti-constipation drugs are common treatments of chronic constipation (Xing and Soffer, 2001 ▶). Because of medications’ side effects and incomplete alleviation of constipation after taking anti-constipation medications, patients use complementary medicine methods (Wang and Yin, 2015 ▶). Additionally, people with a chronic disease, especially ESRD patients, often look for non-invasive alternative therapies such as acupressure to resolve the condition (Cho and Tsay, 2004 ▶). Acupressure is a non-invasive procedure that applies Chinese massage to stimulate acupressure point of the human body (Chen et al., 1999 ▶) and is considered a clinical and comprehensive nursing intervention. Acupressure has advantages such as cost efficacy, lack of complications, no need for special tools, and the ease of learning for the patients and their accompanying persons (Maa, 2005 ▶). Acupressure improves intestinal motility and the function of the digestive system (Tsai, 2011 ▶). Many studies have found that acupressure is effective in improving some symptoms including sleep quality (Tsay et al., 2003 ▶; Tsay et al., 2004 ▶; Tsai, 2011 ▶), fatigue and depression in ESRD patients (Cho and Tsay, 2004 ▶), and thirst in hemodialysis patients (Yang et al., 2010 ▶). But no research was designed to show the effect of acupressure on constipation in ESRD patients. Only one systematic review has shown that application of acupressure relieved chronic constipation in individuals with cerebrovascular accidents, and orthopedics problem, as well as elderlies and people with long-term constipation without specific cause (Fang et al., 2012 ▶), which supports the presumed positive effect. With regard to these few evidence, we hypothesized that acupressure may have a positive effect on mean number of defecation and stool quality in ESRD patients.

The aim of this study was to investigate the effects of acupressure on constipation in patients undergoing hemodialysis. 

## Materials and Methods

We conducted this randomized double-blind placebo-controlled clinical trial from September 2014 to December 2014. Study population included all ESRD patients undergoing chronic hemodialysis in three hemodialysis centers of Talghani, Shahid beheshti, and Imam Khomeini hospitals in Mazandaran, Iran. The inclusion criteria were: (a) age of 18-70 years, (b) diagnosis of ERSD, (c) ESRD subjects routinely receiving 4 hr morning maintenance hemodialysis three times a week, for at least 6 months, (d) good mental health status, with no dementia, (e) ability to communicate in Persian, (f) no limb amputation, (g) not having skin diseases, heart failure, or cerebrovascular accidents, with no history of gastrointestinal disorders such as irritable bowel syndrome, rectal prolapse, anal fissure, volvulus and bowel obstruction, (h) being diagnosed with constipation under the Rome III diagnostic criteria (Chao et al., 2013 ▶). Exclusion criteria were: lack of interest to continue the study, being transferred to intensive care units for any reason, death, transplantation, and traveling. We recruited the eligible patients using the convenience sampling method. A pilot study was carried out on 10 subjects (5 in each group) regarding the average number of defecation in the two groups and the following results were obtained: x̄_1_=13.3, x̄_2_=10.6, S_1_=3.03, and S_2_=3.91. By comparing the group means through sample sizes for two independent samples and considering power of the test (0.8) and confidence level (0.95), a sample size of 26.32 participants was obtained for each group. Considering the probability of dropping off, 35 subjects were enrolled in each primary group by randomization.

The study was a double-blind randomized control trial, so the interviewer and care providers, were unaware of the type of treatment each patient received. The medical documents of 74 ESRD patients who were diagnosed with constipation under the Rome III diagnostic criteria, were assessed. Eligible subjects were reviewed to find couples who were similar in age, sex, and history of hemodialysis to match the effects of these variables on constipation. Each subject in each couple was randomly assigned to either acupressure (receiving acupressure plus routine care), or control (routine care only) groups using simple randomization method. From these 74 subjects who met the initial criteria, only 70 completed the entire trial. Four subjects dropped out from the study for traveling, being transferred to ICU, and transplantation.

**Figure 1:**
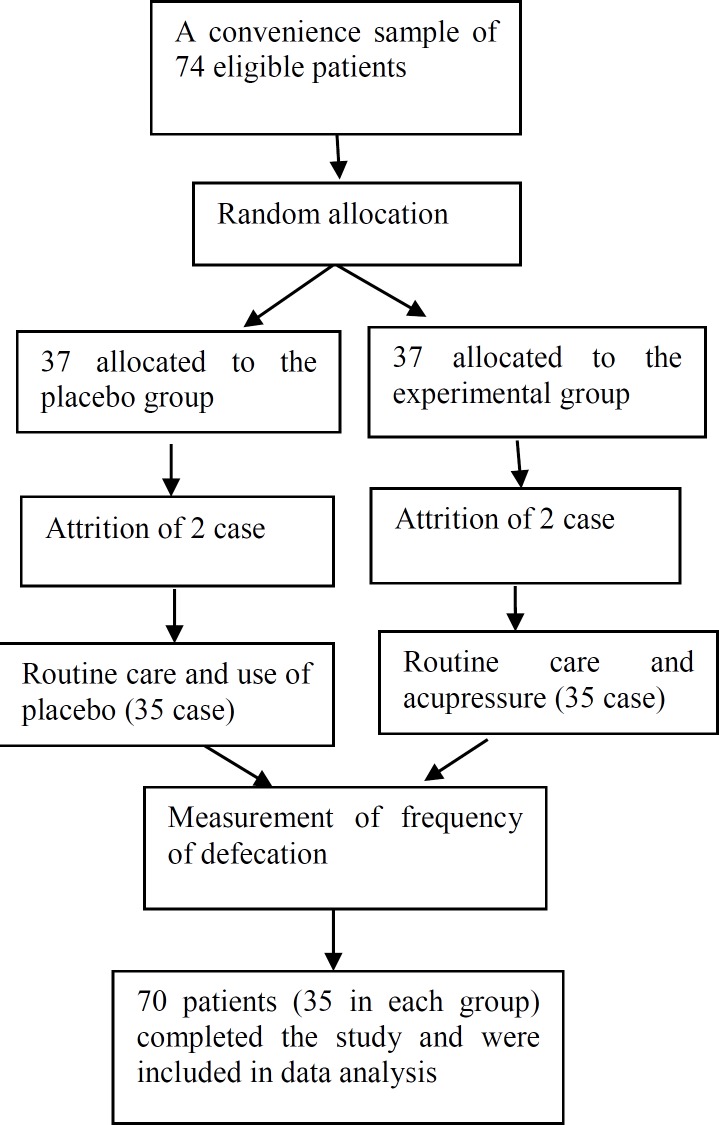
Flow of participants

**Figure 2:**
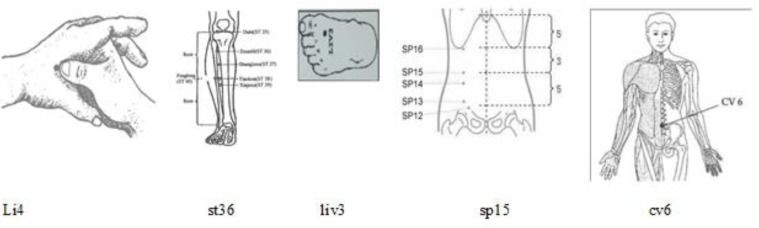
Acupressure point applied to improve constipation (Stein, 2005)

Prior to this study, the researcher and her cooperator were trained to learn acupressure massage under the supervision of a specialist in this field. Intervention was carried out among the subjects regularly undergoing hemodialysis 3 days per week for four consecutive weeks. In the intervention group, the intervention was conducted on the major acupressure points li4, liv3, ST 36, SP15, and cv6 (Stein, 2005 ▶), as presented in Figure 2.

Li4: this points lies between the 1^st^ and 2^nd^ metacarpal bones.

St36: this points is on the legs, 5-6 cm below the knee

Liv3: this point is on the dorsal of fingers of feet between the toe and 4^th^ finger.

Sp15: this point is on the large part of the abdomen horizontal to the navel.

Cv6: this point is below the navel and is the sea of the primary Qi of the body (Standard Acupuncture Nomenclature)

 In the control group, the intervention was carried out with 1 cm distance from the major above-mentioned acupressure points. Each session lasted for 9 min (1min for each acupressure point of li4, liv3, ST 36, and SP15 symmetrically and 1 min for cv6). The amount of needed pressure was practiced by standard scales of 2 to 6 kg, and the reliability of pressure level was confirmed as 100% after 40 times of practice with mean of 3-4 kg on the scales for both researchers’ hands. We assessed frequency of defecation in two groups before and after the study. The study instruments consisted of a demographic questionnaire, and a data sheet for documenting constipation frequency.

 The details about the process of research, goals, and conditions were given to the patients, and consequently, those interested in participation, filled the written consent forms.

Rome III criteria is a questionnaire produced for evaluation of constipation. In relation to these criteria, participants must have presented in the past 6 months with the following symptoms of constipation: (a) rarely having loose stools without the use of laxatives, (b) insufficient evidence of irritable bowel syndrome, and (c) two or more of the following symptoms***:***

1. Straining at defecation at least quarter of the time,

2. Lumpy and/or hard stools at least quarter of the time,

3. A sensation of incomplete evacuation at least quarter of the time,

4. A sensation of anorectal obstruction. Blockage at least quarter of the time, 

5. Manual maneuvers to facilitate defecation at least quarter of the time,

6. Three or fewer defecations per week.

Reliability and validity of the scale was proven in Iran (Sorouri et al., 2010 ▶)

Demographics including age, sex, education level, and marital status, and occupation status, number of children, salary, and physical activity and having history of dialysis treatment, were collected by a questionnaire.

The protocol of the study was approved by ethics committee of Gonabad University of Medical Sciences (No. IR.Gmu.REC.1392) and it was registered in the Iranian clinical trial center (IRCT2015043022027N1). 

**Statistical analysis**

Statistical analyses were performed using the statistical software SPSS version 14 for windows and data were analyzed using descriptive statistics, chi-square test, and independent t-test. One sample Kolmogorov-Smirnov test was conducted to evaluate normality of distribution and non-parametric Mann-Whitney was used for abnormally distributed variables.

## Results

Among 70 subjects, 53.8% were women. The mean age of all subjects was 62.3 years old (for intervention group: 60.3±12.5 years old, and for control group: 64.4±10.6 years old). The two groups were not significantly different in terms of demographic characteristic (Table 1).

**Table 1:** Comparing demographic characteristics between the groups

**Patients’ characteristics**	**Group**	**N**	**mean** **±** **SD**	**Results**
**Age [years: mean (SD)]**	Acupressure	35	60.3±12.5	t=1.22 p=0.23
	Control	35	64.4±10.6	
**Gender (F/M), n (%)**	Acupressure	35	15 (42.86)/20 (57.14)	X^2^=0.27 p=0.61
	Control	35	19 (54.28)/16 (45.72)	
**Marital status (Ma/S), n (%)**	Acupressure	35	31 (88.57)/4 (11.43)	Fisher’s exact test: p=0.24
	Control	35	33 (94.28)/2 (5.72)	
**Education (N/Y), n (%)**	Acupressure	35	17 (48.57)/18 (51.43)	X^2^=1.09 p=0.39
	Control	35	21 (60)/14 (40)	

F, female; M, male; Ma, married; S, single; N, no; Y, yes; n, number;

The independent t-test was used to compare the mean number of defecation per week in 4 weeks after the beginning of intervention in the two groups (Table 2).

**Table 2:** Comparing the mean number of defecation per week in 4 weeks after the beginning of intervention

**Time of intervention**	**Group**	**N**	**Mean** **±** **SD**	**Results**
Weak 1	Acupressure	35	2.73±1.11	z=1.36 p=0.17
	Control	35	2.36±0.81	
Weak 2	Acupressure	35	3.16±1.28	z= 2.48 p=0.01
	Control	35	2.46±1.16	
Weak 3	Acupressure	35	3.73±1.31	z=3.47 p=0.001
	Control	35	2.46±1.25	
Weak 4	Acupressure	35	4.03±1.80	z=2.68 p=0.007
	Control	35	2.76±1.54	

The independent t-test was used for comparing of frequency of defecation between the two groups before and after the intervention. The results showed significant differences between the two groups (Table 3). 

**Table 3:** Comparing the mean number of defecation before and after the intervention

**Results**	**Group**	**N**	**mean** **±** **SD**	**Results**
**One week before** ** beginning of intervention**	Acupressure	35	2.33±0.95	Z=0.20 p=0.84
	Control	35	2.40±1.03	
**4 weeks after beginning of intervention**	Acupressure	35	13.73±3.63	Z=3.55 p<0.001
	Control	35	10.06±3.77	

Covariance analysis was done to compare the mean frequency of defecation between the two groups after adjusting for physical activity (Table 4).

**Table 4:** Comparing the mean frequency of defecation between the two groups after adjusting for physical activity by covariance analysis

**Variable**	**RMSE**	**F**	**P**
**Group**	144.33	10.65	0.002
**Physical activity**	25.63	1.89	0.17

RMSE, Root Mean Squared Error;

## Discussion

The results of this study were consistent with those of previous research on the effect of acupressure on improving gastrointestinal function. Previous studies showed that acupressure has a positive effect on frequency of bowel sounds and movement, the time to first flatus passage, first liquid intake but not the number of defecation per week and stool consistency as diagnostic criteria for constipation, and not in the ESRD patients. For example, Fang et al. conducted an RCT employing acupressure and routine care for patients with acute coronary syndrome and showed that using acupressure on the first admission at acupressure points ST36, T-6, LI4, and SP-6, improved and prevented constipation in these patients. Fang et al. study was similar to our study in terms of using ST36 and LI4 points, but the time and duration of acupressure was unclear (Fang et al., 2012 ▶). Another RCT by Hsing et al. on long-term care patients, showed that performing acupressure at acupressure points ST-25, ST36 and CV12 two minute per day for 15 days improved bowel movement in these patients. study was similar to ours with regard to using ST36 point but they had longer duration of intervention; however, we obtained similar results within a shorter period of time 1 minute per day for 12 non-consecutive days (Tsai, 2011 ▶). 

The effectiveness of ST-36 (Zusanli) acupressure on recovery of post-operative gastrointestinal functions in patients with colorectal cancer was evaluated. In this regard, Chao et al. performed an RCT on 60 patients with colorectal cancer who had undergone abdominal surgery (Chao et al., 2013 ▶). In this study, patients were randomly assigned to two groups, the ST-36 acupressure group and a sham acupressure group. Patients in the ST-36 group received acupressure in a three-minute cycle performed three times per day during the five days after surgery. Patients in the control group received routine postoperative care and sham acupressure. Generalized estimating equations (GEEs) were used to gauge longitudinal effects of the two groups of patients. Patients who received acupressure had significantly earlier flatus passage and time to liquid intake as compared to patients in the control group. But no significant differences were found in the first time to solid intake and defecation between the sham acupressure group and the acupressure group. Chao study showed acupressure on ST-36 was able to shorten the time to first flatus passage, oral liquid intake, and improve gastrointestinal function in patients after abdominal surgery. The number of acupressure point and duration of intervention were less than those used in our study. Therefore, effective of acupressure in our study was more than from other studies. Also, acupressure procedure was done in a one to ten -minute cycle performed during the two weeks decreased constipation by increasing bowel movements in patients with stroke (Tsay and Chen, 2003 ▶). Application of acupressure on acupressure points LI4, LI11 and TE performed for 6-10 minutes per day for two weeks, increased bowel movements and decreased constipation severity (Tsay et al., 2004 ▶). In the two studies mentioned above, patients were not randomly selected, that may cause errors in the interpretation of results. There were studies which examined different approaches to improve constipation. For example, physical therapy for constipation related to puborectalis dyssynergia improved the severity of constipation and quality of life (Tsay et al., 2003 ▶). Acupressure increases endorphins levels in the brain that cause muscle relaxation, pain relief, and comfort (Sun et al., 2010 ▶). So, it is likely that this mechanism helps to reduce constipation.

The present study was the first one performed in Iran in hemodialysis patients suffering from constipation. Nevertheless, the present study had the following limitations: Confounding factors such as diet, addiction, physical activity and use of constipation-inducing drugs existed but their effects were reduced by adjustment. 

This was a two-group study with a rather small sample size. Conducting studies with larger sample sizes is recommended. Moreover, investigating the effects of acupressure using on other acupressure points, is recommended.

Effective constipation management using acupressure could help nurses play an important role in providing effective care to patients undergoing hemodialysis and minimizing adverse effects associated with constipation medications. So, this method can be used as a complementary treatment for improvement of constipation in these patients.

The results of this study showed that acupressure can effectively improve constipation in ESRD patients. Acupressure techniques are non-invasive, safe and effective approaches. Patients and their families could be easily trained to practice acupressure for relief of constipation. Assessment of ESRD patients’ constipation can be a beneficial part of nursing practice, and clinicians may consider providing acupressure as alternative methods for decreasing constipation in these patients and it can decrease adverse health outcomes and improve their quality of life. 

## References

[B1] Andrews CN, Storr M (2011). The pathophysiology of chronic constipation. Can J Gastroenterol Hepatol.

[B2] Atashpeikar S, Jalilazar T, Heidarzade M (2012). Self-care ability in hemodialysis patients. J Caring Sci.

[B3] Chao H, Miao SJ, Liu PF, Lee HH, Chen YM, Yao CT, Chou HL (2013). The beneficial effect of ST-36 (Zusanli) acupressure on postoperative gastrointestinal function in patients with colorectal cancer. Oncol Nurs Forum.

[B4] Chen M, Lin LC, Wu SC, Lin JG (1999). "The effectiveness of acupressure in improving the quality of sleep of institutionalized residents. J Gerontol A Biol Sci Med Sci.

[B5] Cho YC, Tsay SL (2004). "The effect of acupressure with massage on fatigue and depression in patients with end-stage renal disease. J Nurs Res.

[B6] Fang Y-Y, Wang P-L, Tsai C-M, Hsieh M-H (2012). Application of acupressure as a constipation intervention method in patients with acute coronary artery diseases: a systematic review Sigma theta tau internationals 23rd international nursing research congress. Brisbane.

[B7] Hammer J, Osterreicher C, Hammer K, Koch U, Traiendi O, Kovarik J (1998). Chronic gastrointestinal symptoms in hemodialysis patients. Wien Klin Wochenschr.

[B8] Lehnhardt A, Kemper MJ (2011). Pathogenesis, diagnosis and management of hyperkalemia. Pediatric nephrology.

[B9] Lew S (2001). The digestive tract.

[B10] Maa S (2005). Application of acupressure in nursing practice. Hu Li Za Zhi.

[B11] Murtagh FE, Addington-Hall J, Higginson IJ (2007). The prevalence of symptoms in end-stage renal disease: a systematic review. Adv Chronic Kidney Dis.

[B12] Rogers J (2011). How to manage chronic constipation in adults. Nursing times.

[B13] Sorouri M, Pourhoseingholi MA, Vahedi M, Safaee A, Moghimi-Dehkordi B, Pourhoseingholi A, Habibi M, Zali MR (2010). Functional bowel disorders in Iranian population using Rome III criteria. Saudi J Gastroenterol.

[B14] Stark ME (1999). Challenging problems presenting as constipation. Am J Gastroenterol.

[B15] Stein A (2005). Acupressure guide: alleviate headaches, neck and joint pain, anxiety attacks and other ailments, author house.

[B16] Sun JL, Sung MS, Huang MY, Cheng GC, Lin CC (2010). Effectiveness of acupressure for residents of long-term care facilities with insomnia: a randomized controlled trial. Int J Nurs Stud.

[B17] Tsai H (2011). The Effectiveness of acupressure on improving bowel movement in long-term care patient (Thesis).

[B18] Tsay SL, Chen ML (2003). Acupressure and quality of sleep in patients with end-stage renal disease—a randomized controlled trial. Int J Nurs Stud.

[B19] Tsay SL, Cho YC, Chen ML (2004). Acupressure and transcutaneous electrical acupoint stimulation in improving fatigue, sleep quality and depression in hemodialysis patients. Am J Chin Med.

[B20] Tsay S L, Rong JR, Lin PF (2003). Acupoints massage in improving the quality of sleep and quality of life in patients with end‐stage renal disease. J Adv Nurs.

[B21] Vatarii I (2011). The effect of acupressure on quality of sleep in hemodialysis patients. J Med Sci.

[B22] Wang X, Yin J (2015). Complementary and Alternative Therapies for Chronic Constipation. Evid Based Complement Alternat Med.

[B23] Xing JH, Soffer EE (2001). Adverse effects of laxatives. Diseases of the colon & rectum.

[B24] Yang LY, Yates P, Chinn CC, Kaot K (2010). Effect of acupressure on thirst in hemodialysis patients. Kidney Blood Press Res.

